# Deep Learning Approaches for Imaging-Based Automated Segmentation of Tuberous Sclerosis Complex

**DOI:** 10.3390/jcm13030680

**Published:** 2024-01-24

**Authors:** Xuemin Zhao, Xu Hu, Zhihao Guo, Wenhan Hu, Chao Zhang, Jiajie Mo, Kai Zhang

**Affiliations:** 1 Department of Neurophysiology, Beijing Neurosurgical Institute, Capital Medical University, Beijing 100071, China; zhaoxuemin0220@126.com; 2 Department of Neurosurgery, Beijing Tiantan Hospital, Capital Medical University, Beijing 100071, China; zhu_106786@163.com (X.H.); guozhihao1995@foxmail.com (Z.G.); huwenhan88@163.com (W.H.); babaoriley1@163.com (C.Z.); 3 Department of Neurosurgery, Wuxi Taihu Hospital, Wuxi Clinical College of Anhui Medical University, Wuxi 214000, China

**Keywords:** tuberous sclerosis complex, quantitative metrics, deep learning, convolutional neural network

## Abstract

The present study presents a novel approach for identifying epileptogenic tubers in patients with tuberous sclerosis complex (TSC) and automating tuber segmentation using a three-dimensional convolutional neural network (3D CNN). The study retrospectively included 31 TSC patients whose lesions were manually annotated from multiparametric neuroimaging data. Epileptogenic tubers were determined via presurgical evaluation and stereoelectroencephalography recording. Neuroimaging metrics were extracted and compared between epileptogenic and non-epileptogenic tubers. Additionally, five datasets with different preprocessing strategies were used to construct and train 3D CNNs for automated tuber segmentation. The normalized positron emission tomography (PET) metabolic value was significantly lower in epileptogenic tubers defined via presurgical evaluation (*p* = 0.001). The CNNs showed high performance for localizing tubers, with an accuracy between 0.992 and 0.994 across the five datasets. The automated segmentations were highly correlated with clinician-based features. The neuroimaging characteristics for epileptogenic tubers were demonstrated, increasing surgical confidence in clinical practice. The validated deep learning detection algorithm yielded a high performance in determining tubers with an excellent agreement with reference clinician-based segmentation. Collectively, when coupled with our investigation of minimal input requirements, the approach outlined in this study represents a clinically invaluable tool for the management of TSC.

## 1. Introduction

Tuberous sclerosis complex (TSC) is a rare genetic multi-system disease that causes widespread hamartomas in multiple organ systems, such as the skin, eyes, heart, kidney, and brain [1]. It is typically caused by mutations in the tumor suppressor genes TSC1 or TSC2, which enhances the activation of the mechanistic target of rapamycin (mTOR) and causes excessive cell growth and proliferation [2]. Neurological symptoms such as intellectual impairment, autism, and epilepsy are the most disabling aspects of the disease [3]. Cortical tubers are a key characteristic of TSC in neuroimaging. Cortical tubers in patients with TSC can be categorized into three types [4]. As myelination matures, cystic degeneration and calcification occur, and the appearance of cortical tubers in MRI changes [5]. Cortical tubers are thought to be associated with neurological symptoms in addition to being used to diagnose TSC through imaging.

About 80% of patients with TSC experience epilepsy, which usually begins in the first three years of life [6]. In addition, more than 60% of patients suffer from medically intractable epilepsy, and surgical intervention should be considered. Epilepsy surgery is important for treating patients with TSC. A meta-analysis report showed that 59% of patients achieved seizure-free status after surgery [7]. A long-term multicenter study showed that 51% of patients were seizure-free after surgery at 10-year follow-up [8]. Surgical resection of the epileptogenic tubers and surrounding cerebral cortex can reduce or eliminate seizures and enhance neurodevelopmental outcomes [9]. Consequently, the identification of cerebral tubers, particularly the epileptogenic tubers, is crucial for surgical therapy.

MRI, and particularly fluid-attenuated inversion recovery (FLAIR) imaging, has a high capacity for detecting tubers [10]. However, some subtle cerebral tubers are difficult to identify. Also, conventional MRI cannot differentiate between epileptogenic and non-epileptogenic tubers, posing an additional barrier to identifying surgical candidates. The visual assessment of cerebral tubers might rely on the imaging technique and the proficiency of the neuroradiologist. Data characterization extraction algorithms can transform conventional MRI images into high-dimensional, mineable, and quantitative imaging characteristics. This allows for the acquisition of more information about the pathological changes than through visual analysis alone. Manual tuber labeling is time-consuming and prone to errors, making automated detection crucial for efficient preoperative evaluation. Deep learning algorithms have shown promise in medical image segmentation [11].

The current state of machine learning for cybersecurity presents several challenges and limitations, including issues such as data quality, feature selection, and class imbalance, which warrant careful consideration for the development of innovative solutions [12,13]. To address these challenges, this study utilized diverse datasets and preprocessing protocols to train the model, with the aim of exploring the minimal input requirements for epileptogenic tuber segmentation [14].

The contributions of the present study are as follows: (1) Neuroimaging metrics were quantitatively calculated to determine the epileptogenic tubers for guiding surgical resection. This section would help clinicians identify the corresponding tubers to resect and finally improve the prognosis. (2) The second section involved the construction and validation a three-dimensional convolution neural network model for the automated segmentation of intracranial tubers to improve the efficiency of diagnostics in clinical practice.

## 2. Materials and Methods

### 2.1. Patient Population

The study participants were epilepsy patients seeking treatment at the Epilepsy Center, Beijing Fengtai Hospital and Beijing Tiantan Hospital between January 2015 and December 2020. Subjects included in the study met the diagnostic criteria of TSC according to the International TSC Consensus Group in 2012 (Supplemental Material Content S1) [14]. Four classic central nervous system findings are associated with TSC: cortical and sub-cortical tubers, cerebral white matter heterotopia, subependymal nodules (SENs), and subependymal giant cell astrocytoma (SEGAs) [15].

Clinical assessment included a comprehensive evaluation of seizure history and semiology, neurological examination, video-EEG telemetry, and neuroimaging. The epileptogenic tubers of each patient were determined according to the anatomical-electro-clinical correlation provided by the non-invasive examinations. Stereoelectroencephalography (SEEG) implantation was needed if the epileptogenic zone was unclear. Long-term video-SEEG monitoring was performed to capture the seizures in patients to reveal the location of the SEEG-confirmed epileptogenic tubers for surgical resection [16]. Surgical outcomes were collected through outpatient clinics or telephone interviews and were classified according to the ILAE classification system [17].

This study was approved by the Institutional Review Board of Beijing Tiantan Hospital (KY 2020-052-02) and written informed consent was obtained from all participants.

### 2.2. Neuroimaging Protocols

Multiparametric brain MRI data of patients with TSC were obtained from the same protocol [18,19,20,21,22,23]: three-dimensional (3D) T1-weighted magnetization-prepared rapid acquisition gradient echo (T1WI MPGAGE), 3D T2-weighted fluid-attenuated inversion recovery (T2WI FLAIR), and 2-[^18^F] fluoro-2-deoxy-d-glucose (FDG)-PET scans. The MRI scans were performed using a 3-Tesla magnetic resonance imaging (MRI) scanner (MAGNETOM Verio, Siemens, Erlangen, Germany) with the following parameters: T1WI MPRAGE sequence (repetition time (TR) = 2300 ms, echo time (TE) = 2.53 ms, flip angle = 12 degrees, slice thickness = 1 mm, no gap, voxel size = 1.0 mm × 1.0 mm × 1.0 mm), and T2WI FLAIR sequence (TR = 7000 ms, TE = 80 ms, flip angle = 12 degrees, slice thickness = 1 mm, no gap, voxel size = 1.0 mm × 1.0 mm × 1.0 mm). 

Additionally, all PET scans were obtained while the patients were in the interictal state. The ^18^FDG-PET evaluations were performed under the routine resting conditions using the GE Discovery ST PET-CT system (300 mm field of view, matrix 192 × 192, 3.27 mm slice thickness). The patients were required to rest quietly in a dimly lit room during the 40 min period following intravenous administration of ^18^F-FDG at a mean dose of 310 MBq/70 kg body weight. The ordered subset expectation maximization algorithm (sixteen subsets and six iterations) was then employed for PET data reconstruction, which was normalized for attenuation using the transmission scans from a germanium source. PET scans of patients were acquired within 6 months of epilepsy surgery evaluation and no clinical seizures were reported 6 h prior to or during the PET scans [24,25,26].

### 2.3. Images Preparation

The tuber masks of all included patients were manually drawn and validated by at least two neuroradiologists using MRIcron software (v1.0.20190902, https://www.nitrc.org/projects/mricron, accessed on 27 July 2023). Figure S1 shows a three-dimensional view of the tuber masks. In the training step, the tuber masks were used as the ground truth for the segmentation [22], and resampled to the same voxel size using nearest neighbor resampling. The tubers were annotated as epileptogenic and non-epileptogenic tubers based on the results of presurgical evaluation or conformation from SEEG recordings, respectively. The results from the SEEG-confirmed epileptogenicity could provide robust evidence for the final results. In this analysis, the tubers without SEEG electrode recordings were excluded.

**Figure 1:**
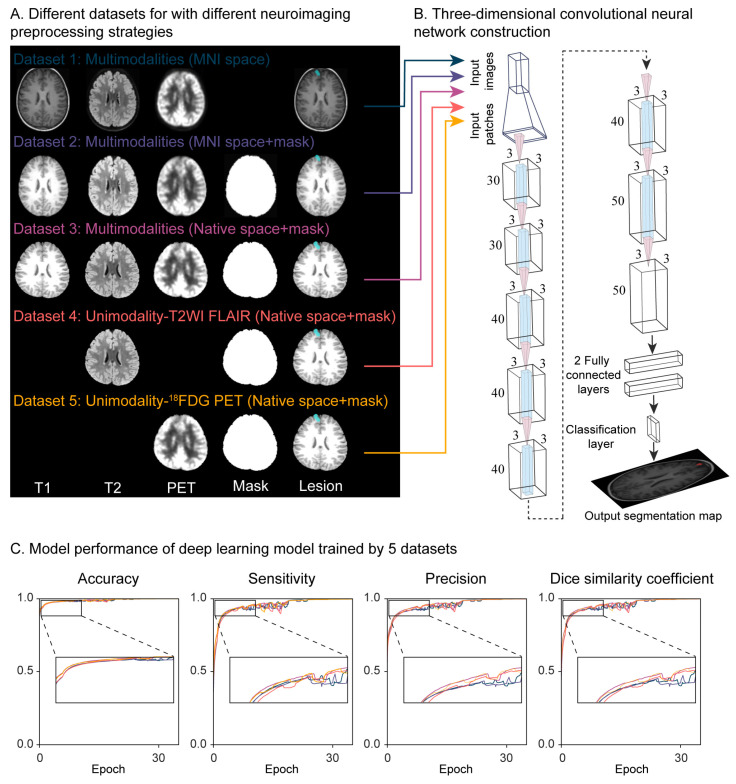
Deep learning architecture and performance for tuber segmentation. (**A**) Different training datasets with 5 different procedures. Rows 1–3 indicate the multimodality images used for training to investigate the advantage of the full utilization of complete information, and rows 4–5 indicate the unimodality images used to explore the advantage of minimum image scanning and time saving. (**B**) The schematics for the network architecture, adapted for intracranial tuber detection. The number of features in all convolutional layers was 30, 30, 40, 40, 40, 40, 50, and 50, followed by 2 fully connected layers and the tuber probability map. (**C**) Tuber segmentation performance of 5 trained models evaluated for accuracy, sensitivity, precision, and the Dice similarity coefficient. A detailed statistic summary of model performance is shown in Table S4.

The study investigated and compared the model performances trained by different datasets. Data preprocessing methods included the following common procedures: (i) The T1WI MPRAGE images were first reoriented to the anterior commissure–posterior commissure line using the Display tool of statistical parametric mapping 12 (SPM12) software (Wellcome Department of Cognitive Neurology, University College, London, UK) running on Matlab 2021a (MathWorks Inc., Natick, MA, USA); (ii) bias field inhomogeneity correction was performed to minimize B1-induced radiofrequency inhomogeneity; (iii) intensity was standardized to zero-mean and unit variance. Five deep-learning models were trained separately using five datasets with further different preprocessing. This diversity allowed us to explore a range of preprocessing options and identify an optimal protocol that balances simplicity and time efficiency with high performance. Figure 1A shows examples of the datasets used: (i) Dataset 1: multimodalities images spatially normalized to Montreal Neurological Institute (MNI) space; (ii) dataset 2: multimodalities images with MNI normalization and brain mask; (iii) dataset 3: multimodalities images with native space and brain mask; (iv) dataset 4: T2WI FLAIR images with native space and brain mask; (v) dataset 5: ^18^FDG-PET images with native space and brain mask.

### 2.4. Convolutional Neural Network Construction

The three-dimensional convolutional neural network (3D-CNN), DeepMedic [27], was designed and trained for a voxel-wise dual class segmentation of the tuber. The 3D-CNN contains eight convolutional layers (kernel size = 3^3^) for each resolution pathway, followed by two fully connected layers implemented as 1^3^ convolutions and a final classification layer (Figure 1B). Only the normal-resolution pathway was implemented. Patches of the size of 25^3^ voxels were extracted for training using class balancing of 50% of the samples centered on a positive tuberous voxel and 50% centered on a voxel not containing tubers. To generalize the reliability of the network, 31 patients with TSC were randomly shuffled and distributed into three groups (training, validation, and test datasets) in a 5:2:3 ratio to avoid fitting [28,29]. Finally, the training data consisted of 14 patients, while seven patients were used as the validation dataset, and the remaining ten were used as the test dataset.

### 2.5. Statistical Analysis

Quantitative neuroimaging metrics, including normalized tuber volumes, T1 signal value, FLAIR intensity, and PET metabolic value, were compared between epileptogenic or non-epileptogenic tubers using Student’s *t*-test or the non-parametric Mann–Whitney *U* test after a normal distribution test (Lilliefors test). First, analyses across all included patients (31 patients with 207 tubers) were performed to investigate the neuroimaging metrics. However, some patients had single huge tubers, which could affect the statistical power. Therefore, neuroimaging metrics were evaluated mainly between epileptogenic and non-epileptogenic tubers only in the cohort of patients with more than one tuber (25 patients with 201 tubers). Lastly, to obtain more accurate neuroimaging metrics of epileptogenic tubers, a subgroup analysis of SEEG-confirmed epileptogenic tubers in the cohort of patients with SEEG implantation (16 patients with 65 tubers) was performed.

The performance of each 3D CNN model was assessed by measuring the accuracy, sensitivity, precision, and Dice similarity coefficient (DSC). A receiver operating characteristic (ROC) curve was also calculated for each training. The performance measurements across five models were evaluated using the nonparametric Kruskal–Wallis test. Neuroimaging metrics of automated contours and manual segmentation were evaluated using Pearson’s correlation coefficients (*r*), which were calculated for each extracted metric.

Data were analyzed using SPSS software version 26.0 (IBM Corp, Armonk, NY, USA). A two-sided value of *p* < 0.05 was considered statistically significant.

## 3. Results

### 3.1. Demographic Data

Finally, 31 (17 female (54.8%)) of the 44 patients who passed the first screening with complete and high-quality neuroimaging for quantitative neuroimaging metrics comparison and segmentation model development were selected for retrospective analysis. Table 1 shows demographic, imaging, and follow-up data. Of those, the age at onset was 5.8 ± 6.7 years, and the disease lasted 7.8 ± 6.7 years. Subsequent SEEG implantation was performed in 16 patients (51.6%), and epilepsy surgery was performed in 29 patients (93.5%). All the included patients were followed-up after at least one year (43.3 ± 23.5 months). The surgical prognosis of eleven patients was unavailable, and 14 of the remaining 20 patients were seizure-free (ILAE 1: 70%) after epilepsy surgery. 

### 3.2. Quantitative Neuroimaging Metrics Comparison

**Figure 2:**
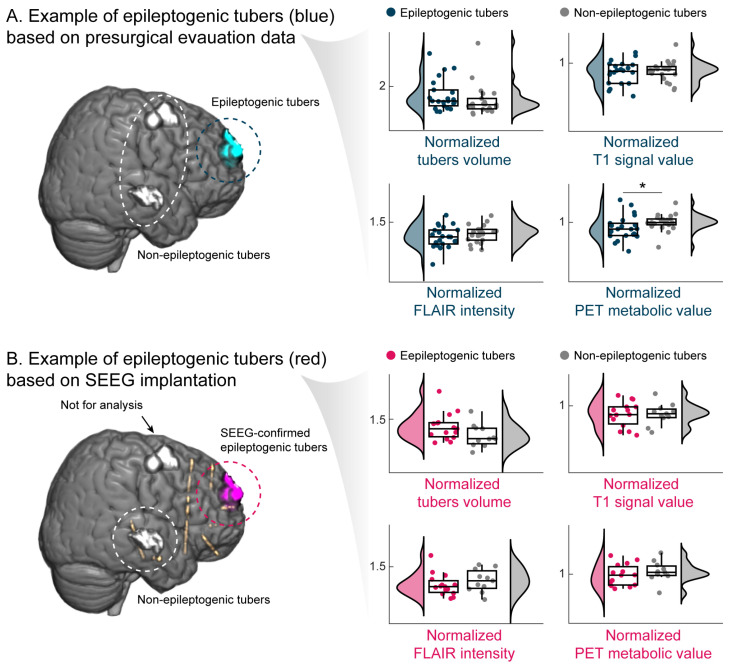
Quantitative neuroimaging metrics comparison. (**A**) Comparison between corresponding and non-corresponding tubers in the cohort of patients with more than 1 tuber (25 patients with 201 tubers). Blue tubers are the corresponding tubers, while the grey tubers are the non-corresponding tubers investigated by the presurgical evaluation. (**B**) Comparison between corresponding and non-corresponding tubers with SEEG recording-confirmed epileptogenicity (14 patients with 65 tubers). Pink tubers are the corresponding tubers, while the grey tubers are the non-corresponding tubers investigated by the SEEG implantation, and the tubers without SEEG electrodes passing were excluded for analysis. *: *p* < 0.05.

The study first evaluated the metrics between epileptogenic and non-epileptogenic tubers across all patients. The epileptogenic tubers were significantly larger (Mann–Whitney *U* = 2.382, *p* = 0.017), and metabolism was lower (Mann–Whitney *U* = −3.274, *p* = 0.001) (Figures S1 and S2 and Table S1). The normalized T1 signal value (Mann–Whitney *U* = −0.264, *p* = 0.792) and normalized FLAIR intensity (independence Student’s *t* = −1.082, *p* = 0.285) showed no significant differences. Then, the metrics between epileptogenic and non-epileptogenic tubers in the cohort of patients with more than one tuber were evaluated. As shown in Figure 2A, only the normalized PET metabolic value of epileptogenic tubers was significantly lower than that of non-epileptogenic tubers (paired Student’s *t* = −2.502, *p* = 0.021), while the other metrics, including the normalized tubers volume (paired Wilcoxon *z* = 1.749, *p* = 0.083), normalized T1 signal value (paired Student’s *t* = −0.905, *p* = 0.376), and normalized FLAIR intensity (paired Student’s *t* = −1.451, *p* = 0.162) showed no significant differences (Table S2).

Furthermore, to better define the epileptogenic tubers, the SEEG-confirmed epileptogenic tubers were analyzed. The results across the four metrics were insignificant (Table S3). However, the trend of a larger volume of tubers and a lower PET metabolism of epileptogenic tubers could be observed (Figure 2B).

### 3.3. Convolutional Neural Network Construction and Performance

Considering the factors of minimum input requirements, less radiation, and time efficiency, the performance of deep learning models trained on five datasets with five different types of neuroimaging preprocessing was investigated. Figure 1C shows the segmentation performance of the five models, plotted as a function of different cut points of CNN’s probabilistic output. The CNN demonstrated near-identical performance curves with extremely high sensitivity and specificity. For the five CNNs, the median, interquartile range (IQR) for accuracy was 0.993 (0.983–0.999), 0.993 (0.982–0.999), 0.992 (0.986–0.999), 0.994 (0.985–0.999), and 0.992 (0.986–0.999); sensitivity was 0.975 (0.937–0.997), 0.974 (0.932–0.996), 0.969 (0.942–0.996), 0.975 (0.938–0.996), and 0.966 (0.938–0.995); precision was 0.972 (0.929–0.996), 0.971 (0.928–0.996), 0.969 (0.944–0.995), 0.975 (0.939–0.995), and 0.967 (0.945–0.995); and DSC was 0.973 (0.933–0.997), 0.973 (0.933–0.997), 0.973 (0.933–0.997), 0.973 (0.933–0.997), and 0.973 (0.933–0.997), respectively. All measurements were not significantly different across the five models (accuracy: Kruskal–Wallis *H* = 0.373, *p* = 0.985; sensitivity: Kruskal–Wallis *H* = 0.224, *p* = 0.994; precision: Kruskal–Wallis *H* = 0.180, *p* = 0.996; DSC: Kruskal–Wallis *H* = 0.209, *p* = 0.995) (Table S4).

Procedures for image preprocessing took approximately 12 min for each patient in datasets 1 and 2, 5 min in dataset 3, and 3 min in datasets 4 and 5. Each deep-learning network was trained for approximately 21 h. The trained networks took approximately 3 min to voxel-wise segment the intracranial tubers for each subject.

### 3.4. Comparison of Automatically Segmented and Clinician-Based Features

**Figure 3:**
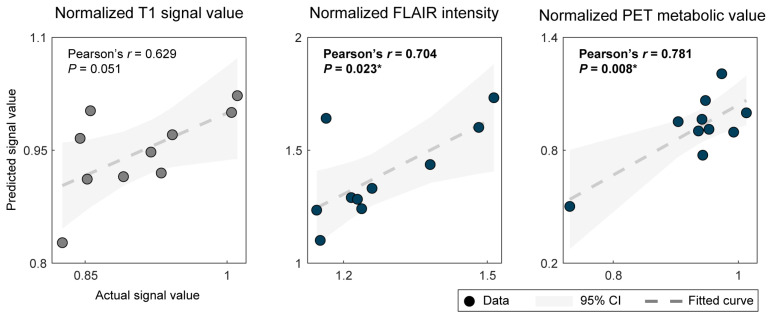
Comparison of clinician-based and automatically segmented metrics: normalized T1 signal value, FLAIR intensity, and PET metabolic value. Pearson correlation coefficients (*r*) are shown in the top left corner of each plot, with dotted lines showing the fitted curve and shaded areas showing the 95% confidence interval. Grey dots indicate non-significance, whereas blue dots and * indicate significant differences.

Figure 3 shows the relationship calculation of quantitative neuroimaging metrics of CNN based on automatically segmented and clinician-based masks. The normalized FLAIR intensity and PET metabolic value of automated segmentation were strongly correlated with the clinician definition, with Pearson’s correlation coefficients of *r* = 0.704 (*p* = 0.023) and *r* = 0.781 (*p* = 0.008), while the normalized T1 signal value showed no significant correlation (Pearson’s *r* = 0.629, *p* = 0.051) (Figure 3).

## 4. Discussion

### 4.1. Strengths

In this study, the neuroimaging characteristics of epileptogenic tubers were calculated and compared for guiding surgical resection. According to the findings of this study, the presence of a large tuber volume and hypometabolism strongly suggests the presence of an epileptogenic zone. To better define epileptogenic tubers for statistical comparison, not only the presurgical evaluation of routine multidisciplinary discussion but also intracranial SEEG-electrophysiological recording was used for further validation. A deep-learning algorithm with high performance for detecting intracranial tubers with an excellent agreement with manual segmentation by clinicians was constructed and validated. Moreover, an optimal neuroimaging preprocessing for model training was explored. A strategy using only native FLAIR images was found, with minimum input requirements, less radiation, and time efficiency.

### 4.2. Quantitative Neuroimaging Metrics for Epileptogenic Tubers

The preoperative assessment of epileptogenic tubers in TSC patients remains challenging. If noninvasive preoperative examination could be utilized to localize epileptogenic tubers, more individuals with TSC might benefit from surgical resection. Several previous studies have used neuroimaging techniques to differentiate epileptogenic from non-epileptogenic tubers. Previous studies suggested that measuring the maximum apparent diffusion coefficient (ADC) and radial diffusivity (RD) with diffusion tensor imaging (DTI) in both tuber and peritubular tissues can identify epileptogenic tubers [30]. The orientation dispersion index (ODI), derived from neurite orientation dispersion and density imaging (NODDI), can also detect epileptogenicity.

This study extracted four quantitative neuroimaging metrics, including normalized tuber volumes, T1 signal value, FLAIR intensity, and PET metabolic value. In line with previous studies, epileptogenic tubers were larger than non-epileptogenic ones [29,31]. The tuber volume was positively correlated with DTI changes in the corpus callosum and internal capsules [32]. It indicated that a larger tuber volume was more likely to be associated with a greater tuber load, which causes more severe neurological symptoms.

Another promising finding was that the metabolism in epileptogenic tubers was lower than in non-epileptogenic tubers. PET plays an important role in understanding brain development and neurodevelopmental diseases. Additionally, PET scans are highly effective for detecting epileptogenic zones and guiding surgical resection. Different PET tracers exhibit distinct characteristics in TSC tubers.

^18^FDG-PET frequently demonstrates hypometabolism in and around epileptogenic and non-epileptogenic tubers, which often extends beyond structural abnormalities. This is thought to be caused by a reduction in the number of neurons and a simplified dendritic pattern of tubers [33]. Some authors recommended using FDG-PET/MRI coregistration and choosing the tubers with the most hypometabolism relative to their MRI size to identify the epileptogenic tubers [34]. Nevertheless, in a later study at the same center, 16 of 28 TSC patients could not be localized by the FDG-PET/MRI coregistration because no single tuber or group of tubers exhibited increased hypometabolism relative to the actual MRI volume [35]. A recent study delineated the correlations between G-protein-coupled receptor 30 (GPR30) and ^18^FDG-PET values in female patients with TSC [36]. PET uptake values were useful in recognizing epileptogenic tubers in female patients with TSC in a non-invasive way. α-[^11^C]-methyl-l-tryptophan (AMT)-PET scans indicated higher AMT uptake in TSC tubers, and the level of AMT uptake may distinguish between epileptogenic and non-epileptogenic tubers [37,38].

The quantitative neuroimaging metrics of the epileptogenic tubers were analyzed using a layer-by-layer approach. First, the per-lesion analysis of all patients revealed substantially larger epileptogenic tubers with a lower metabolic value. However, some patients may have only one giant tuber, which may induce bias. Consequently, patients with more than one tuber were analyzed, revealing that only the normalized PET metabolic value of epileptogenic tubers was significantly lower than that of non-epileptogenic tubers. SEEG is usually considered the gold standard for localizing the seizure onset zone. Therefore, tuber metrics were analyzed in the subgroup of patients implanted with SEEG. Although the results were statistically insignificant, the trends of a larger volume of tubers and a lower PET metabolism of epileptogenic tubers were observed. Further study with more powerful analyses and a larger sample is warranted to identify epileptogenic tubers noninvasively and more precisely.

### 4.3. Automated Tuber Segmentation

This study took a pioneering initiative to use a CNN model for automated tuber segmentation. In many neurological disorders, the findings of brain MRI can be considered a reliable biomarker that reflects disease severity. Despite the fact that cortical tubers in TSC are a prominent neurophysiological hallmark, their role in pathophysiology remains unknown. Patients with TSC frequently exhibit neurologic symptoms, but some patients may be asymptomatic even when they have numerous tubers [3].

However, the accurate detection of cortical tubers is crucial for the diagnosis and further treatment of TSC patients. On brain MRI, cortical tubers in TSC appear populous and heterogeneous, and accurate classification of tubers via visual analysis is usually dependent on the experience of the radiologist. In previous studies, TSC tubers or other epileptogenic lesions have been detected using morphometric MRI or surface-based analysis based on quantitative imaging feature extraction [21,39]. Although these methods are powerful, they rely on manual feature extraction for visualization or model training.

With the development of deep learning technology, more and more studies are using deep learning methods to segment brain lesions [30]. CNNs have been recognized for their superior performance ability to learn robust features and predict corresponding labels when given inputs directly in a supervised end-to-end manner.

The results of the deep learning models depend on how the input data are represented. Five deep learning models were built and trained using five different neuroimaging preprocessing methods. The model with minimum input requirements, less radiation, and time-efficiency was investigated. All CNNs demonstrated near-identical performance curves with extremely high sensitivity and specificity. The five CNNs were compared for accuracy, sensitivity, precision, and DSC, and the difference across the five models was insignificant. The CNN trained using T2WI FLAIR images with native space and a brain mask displayed a remarkable performance, achieving an accuracy of 0.994 and a sensitivity of 0.975. The current results demonstrate optimal neuroimaging preprocessing for model training. Also, the results reveal that T2WI FLAIR images in the native space alone can achieve good segmentation results without spatial normalization or metabolic imaging.

### 4.4. Limitations

The epileptogenic tubers were unambiguously defined only if the patient received surgery and remained seizure-free in the last follow-up [14]. Unfortunately, surgical resection and resultant data on seizure freedom were unavailable for some of our patients. Therefore, the present study elected to derive a “suspected” epileptogenic tuber using presurgical evaluation information. For example, long-term video EEG monitoring was performed to record the interictal and ictal EEG patterns with the simultaneous semiology evolution. In addition to the electrophysiological findings, epileptologists also interpreted the MRI findings. Finally, the tubers with well-defined anatomo-electro-clinical correlations were considered as epileptogenic tubers. To minimize this limitation, the epileptogenic tubers were further defined according to the SEEG recording, and the tubers without SEEG electrode coverage were excluded to make the results more credible.

## 5. Conclusions

Neuroimaging features for epileptogenic tubers were demonstrated based on information from the presurgical evaluation information and SEEG recording, which increased surgical confidence in clinical practice. The validated deep learning detection algorithm yielded high performance in tuber determination with an excellent agreement with reference clinician-based segmentation. Collectively, when coupled with our investigation of minimal input requirements, the approach outlined in this study represents a clinically invaluable tool for the management of TSC.

## Figures and Tables

**Table 1:** Demographic and clinical data of patients with tuberous sclerosis complex.

No.	Sex	Age at Epilepsy Onset (yrs)	Age at Presurgical Evaluation (yrs)	Duration of Disease (yrs)	Number of Tubers	SEEG Implantation	Surgery	Neuroimaging Representation	Follow-Up	Prognosis
1	F	4	6	2	1	N	Lh. P	CTs, RML	82	NA
2	F	9	19	10	9	Y	Rh. F	CTs, RML, SENs	86	ILAE 1
3	M	9	10	1	1	Y	Lh. F	CTs, WMH	81	NA
4	M	2	14	12	6	Y	Rh. F	CTs, RML, SENs	69	NA
5	F	10	13	3	4	Y	Lh. F	CTs, RML	77	ILAE 4
6	M	2	16	14	8	Y	Rh. F	CTs, RML	70	ILAE 1
7	M	4	8	4	5	Y	Lh. F	CTs, RML	57	ILAE 4
8	F	6	14	8	1	N	Lh. F	CTs, SEGAs	58	ILAE 3
9	F	1	7	6	22	Y	Rh. F-I	CTs, RML, SENs	59	ILAE 4
10	M	1	6	5	1	N	Rh. F	CTs	64	NA
11	F	0.5	8	7.5	12	Y	Rh. F	CTs, RML	60	ILAE 1
12	F	12	23	11	7	N	Rh. F	CTs	51	NA
13	M	3	30	27	2	N	Rh. F	CTs, RML	52	NA
14	F	10	27	17	21	N	Lh. P-O	CTs, RML, SENs	49	ILAE 5
15	F	18	24	6	7	N	Rh. F	CTs, RML, SENs	36	NA
16	M	1	2	1	3	Y	Rh. I	CTs	36	ILAE 1
17	F	4	16	12	9	N	Lh. F	CTs, RML	35	ILAE 1
18	F	8	12	4	1	Y	Rh. I	CTs	38	ILAE 1
19	F	3.8	4.1	0.3	3	N	Lh. F	CTs	20	ILAE 1
20	M	5.7	8.9	3.2	9	Y	Lh. F	CTs, SENs	21	ILAE 1
21	M	0.5	25	24.5	5	Y	Medication	CTs, RML	19	NA
22	M	2	12	10	10	N	Medication	CTs, SENs	18	NA
23	F	1.2	6.6	5.4	8	N	VNS	CTs, SENs	17	NA
24	F	2	4	2	6	N	Lh. F	CTs, SENs	49	NA
25	F	1	3	2	14	N	Rh. F	CTs, RML, SENs	45	ILAE 4
26	M	0.2	8	7.8	2	Y	Lh. P	CTs	18	ILAE 1
27	F	5	22	17	1	N	Lh. P-O	CTs	16	ILAE 1
28	M	2.5	3.5	1	4	Y	Rh. F	CTs, RML	13	ILAE 1
29	F	32	38	6	4	N	Lh. F	CTs, RML	16	ILAE 1
30	M	3.3	5	1.8	2	Y	Rh. F	CTs	15	ILAE 1
31	M	15	24	9	19	Y	Rh. F-P	CTs, RML	16	ILAE 1

SEEG: stereoelectroencephalography; F: female; M: male; Lh: left side; Rh: right side; F: frontal lobe; P: parietal lobe; I: insular lobe; O: occipital lobe; VNS: vagus nerve stimulation; CTs: cortical and sub-cortical tubers; RML: radial migration lines; SENs: subependymal nodules; SEGAs: subependymal giant cell astrocytoma; NA: not available.

## Data Availability

The data that support the findings of this study are available from the corresponding author upon reasonable request.

## Supplementary Materials

The following supporting information can be downloaded at: https://www.mdpi.com/article/10.3390/jcm13030680/s1, Content S1: Diagnostic criteria for tuberous sclerosis complex (TSC) 2012 [15]; Table S1: Statistic of multimodal neuroimaging characteristics of tubers across all subjects; Table S2: Statistic of multimodal neuroimaging characteristics of tubers across all subjects with several tubers; Table S3: Statistic of multimodal neuroimaging characteristics of tubers across all subjects with SEEG implantation; Table S4: Statistic summary of model performance trained by 5 different datasets; Figure S1: Visualization of intracranial tubers distribution across all 31 included patients. Blue tubers were the epileptogenic tubers, while the yellow tubers were the non-epileptogenic tubers investigated by the presurgical evaluation; Figure S2: Multimodal quantitative neuroimaging metrics comparison of intracranial tubers across all 31 included. Detail statistical analysis was shown in Table S3. *: significant difference.

## References

[1] Curatolo P., Moavero R., de Vries P.J. (2015). Neurological and neuropsychiatric aspects of tuberous sclerosis complex. Lancet Neurol..

[2] Salussolia C.L., Klonowska K., Kwiatkowski D.J., Sahin M. (2019). Genetic Etiologies, Diagnosis, and Treatment of Tuberous Sclerosis Complex. Annu. Rev. Genom. Hum. Genet..

[3] Curatolo P., Specchio N., Aronica E. (2022). Advances in the genetics and neuropathology of tuberous sclerosis complex: Edging closer to targeted therapy. Lancet Neurol..

[4] Gallagher A., Grant E.P., Madan N., Jarrett D.Y., Lyczkowski D.A., Thiele E.A. (2010). MRI findings reveal three different types of tubers in patients with tuberous sclerosis complex. J. Neurol..

[5] Vaughn J., Hagiwara M., Katz J., Roth J., Devinsky O., Weiner H., Milla S. (2013). MRI Characterization and Longitudinal Study of Focal Cerebellar Lesions in a Young Tuberous Sclerosis Cohort. Am. J. Neuroradiol..

[6] Chu-Shore C.J., Major P., Camposano S., Muzykewicz D., Thiele E.A. (2010). The natural history of epilepsy in tuberous sclerosis complex. Epilepsia.

[7] Zhang K., Hu W.-H., Zhang C., Meng F.-G., Chen N., Zhang J.-G. (2013). Predictors of seizure freedom after surgical management of tuberous sclerosis complex: A systematic review and meta-analysis. Epilepsy Res..

[8] Liu S., Yu T., Guan Y., Zhang K., Ding P., Chen L., Shan Y., Guo Q., Liu Q., Yao Y. (2020). Resective epilepsy surgery in tuberous sclerosis complex: A nationwide multicentre retrospective study from China. Brain.

[9] Shahid A. (2013). Resecting the epileptogenic tuber: What happens in the long term?. Epilepsia.

[10] Pinto Gama H.P., da Rocha A.J., Braga F.T., da Silva C.J., Maia A.C.M., de Campos Meirelles R.G., Mendonça do Rego J.I., Lederman H.M. (2006). Comparative analysis of MR sequences to detect structural brain lesions in tuberous sclerosis. Pediatr. Radiol..

[11] Shaukat K., Luo S., Varadharajan V. (2023). A novel deep learning-based approach for malware detection. Eng. Appl. Artif. Intell..

[12] Shaukat K., Luo S., Varadharajan V., Hameed I.A., Chen S., Liu D., Li J. (2020). Performance comparison and current challenges of using machine learning techniques in cybersecurity. Energies.

[13] Shaukat K., Luo S., Varadharajan V., Hameed I.A., Xu M. (2020). A survey on machine learning techniques for cyber security in the last decade. IEEE Access.

[14] Park D.K., Kim W., Thornburg O.S., McBrian D.K., McKhann G.M., Feldstein N.A., Maddocks A.B., Gonzalez E., Shen M.Y., Akman C. (2022). Convolutional neural network-aided tuber segmentation in tuberous sclerosis complex patients correlates with electroencephalogram. Epilepsia.

[15] Northrup H., Krueger D.A., Northrup H., Krueger D.A., Roberds S., Smith K., Sampson J., Korf B., Kwiatkowski D.J., Mowat D. (2013). Tuberous Sclerosis Complex Diagnostic Criteria Update: Recommendations of the 2012 International Tuberous Sclerosis Complex Consensus Conference. Pediatr. Neurol..

[16] Wang M.X., Segaran N., Bhalla S., Pickhardt P.J., Lubner M.G., Katabathina V.S., Ganeshan D. (2021). Tuberous Sclerosis: Current Update. Radiogr. Rev. Publ. Radiol. Soc. N. Am. Inc..

[17] Guo Z., Zhao B., Toprani S., Hu W., Zhang C., Wang X., Sang L., Ma Y., Shao X., Razavi B. (2020). Epileptogenic network of focal epilepsies mapped with cortico-cortical evoked potentials. Clin. Neurophysiol..

[18] Wieser H.G., Blume W.T., Fish D., Goldensohn E., Hufnagel A., King D., Sperling M.R., Lüders H., Pedley T.A., Commission on Neurosurgery of the International League Against Epilepsy (ILAE) ILAE Commission Report (2001). Proposal for a new classification of outcome with respect to epileptic seizures following epilepsy surgery. Epilepsia.

[19] Guo Z., Mo J., Zhang C., Zhang J., Hu W., Zhang K. (2022). Brain-clinical signatures for vagus nerve stimulation response. CNS Neurosci. Ther..

[20] Mo J., Dong W., Cui T., Chen C., Shi W., Hu W., Zhang C., Wang X., Zhang K., Shao X. (2022). Whole-brain metabolic pattern analysis in patients with anti-leucine-rich glioma-inactivated 1 (LGI1) encephalitis. Eur. J. Neurol..

[21] Mo J., Liu Z., Sun K., Ma Y., Hu W., Zhang C., Wang Y., Wang X., Liu C., Zhao B. (2019). Automated detection of hippocampal sclerosis using clinically empirical and radiomics features. Epilepsia.

[22] Mo J., Zhang J., Hu W., Luo F., Zhang K. (2021). Whole-brain morphological alterations associated with trigeminal neuralgia. J. Headache Pain.

[23] Mo J., Zhang J., Hu W., Sang L., Zheng Z., Zhou W., Wang H., Zhu J., Zhang C., Wang X. (2022). Automated Detection and Surgical Planning for Focal Cortical Dysplasia with Multicenter Validation. Neurosurgery.

[24] Mo J., Zhang J., Hu W., Shao X., Sang L., Zheng Z., Zhang C., Wang Y., Wang X., Liu C. (2022). Neuroimaging gradient alterations and epileptogenic prediction in focal cortical dysplasia IIIa. J. Neural Eng..

[25] Guo Z., Zhang C., Wang X., Liu C., Zhao B., Mo J., Zheng Z., Shao X., Zhang J., Zhang K. (2022). Is intracranial electroencephalography mandatory for MRI-negative neocortical epilepsy surgery?. J. Neurosurg..

[26] Mo J., Wei W., Liu Z., Zhang J., Ma Y., Sang L., Hu W., Zhang C., Wang Y., Wang X. (2021). Neuroimaging Phenotyping and Assessment of Structural-Metabolic-Electrophysiological Alterations in the Temporal Neocortex of Focal Cortical Dysplasia IIIa. J. Magn. Reson. Imaging JMRI.

[27] Mo J., Zhang J., Hu W., Sang L., Shao X., Zhang C., Zhang K. (2022). Metabolism and Intracranial Epileptogenicity in Temporal Lobe Long-Term Epilepsy-Associated Tumor. J. Clin. Med..

[28] Alves A.F.F., de Miranda J.R.A., Reis F., de Souza S.A.S., Alves L.L.R., de Feitoza L.M., de Souza de Castro J.T., de Pina D.R. (2020). Inflammatory lesions and brain tumors: Is it possible to differentiate them based on texture features in magnetic resonance imaging?. J. Venom. Anim. Toxins Trop. Dis..

[29] Dietterich T. (1995). Overfitting and undercomputing in machine learning. ACM Comput. Surv. CSUR.

[30] Kamnitsas K., Ledig C., Newcombe V.F.J., Simpson J.P., Kane A.D., Menon D.K., Rueckert D., Glocker B. (2017). Efficient multi-scale 3D CNN with fully connected CRF for accurate brain lesion segmentation. Med. Image Anal..

[31] Yogi A., Hirata Y., Karavaeva E., Harris R.J., Wu J.Y., Yudovin S.L., Linetsky M., Mathern G.W., Ellingson B.M., Salamon N. (2015). DTI of tuber and perituberal tissue can predict epileptogenicity in tuberous sclerosis complex. Neurology.

[32] Shao X., Zhang X., Xu W., Zhang Z., Zhang J., Guo H., Jiang T., Zhang W. (2021). Neurite orientation dispersion and density imaging parameters may help for the evaluation of epileptogenic tubers in tuberous sclerosis complex patients. Eur. Radiol..

[33] Chou I.-J., Lin K.-L., Wong A.M., Wang H.-S., Chou M.-L., Hung P.-C., Hsieh M.-Y., Chang M.-Y. (2008). Neuroimaging correlation with neurological severity in tuberous sclerosis complex. Eur. J. Paediatr. Neurol..

[34] Yogi A., Hirata Y., Linetsky M., Ellingson B.M., Salamon N. (2022). Qualitative and quantitative evaluation for the heterogeneity of cortical tubers using structural imaging and diffusion-weighted imaging to predict the epileptogenicity in tuberous sclerosis complex patients. Neuroradiology.

[35] Simao G., Raybaud C., Chuang S., Go C., Snead O.C., Widjaja E. (2010). Diffusion Tensor Imaging of Commissural and Projection White Matter in Tuberous Sclerosis Complex and Correlation with Tuber Load. Am. J. Neuroradiol..

[36] Wang Z., Huang K., Yang X., Shen K., Yang L., Ruan R., Shi X., Wang M., Zhu G., Yang M. (2021). Downregulated GPR30 expression in the epileptogenic foci of female patients with focal cortical dysplasia type IIb and tuberous sclerosis complex is correlated with ^18^F-FDG PET-CT values. Brain Pathol..

[37] Chugani H.T., Luat A.F., Kumar A., Govindan R., Pawlik K., Asano E. (2013). α-[11C]-Methyl-L-tryptophan-PET in 191 patients with tuberous sclerosis complex. Neurology.

[38] Rubí S., Costes N., Heckemann R.A., Bouvard S., Hammers A., Martí Fuster B., Ostrowsky K., Montavont A., Jung J., Setoain X. (2013). Positron emission tomography with α-[^11^C]methyl-l-tryptophan in tuberous sclerosis complex-related epilepsy. Epilepsia.

[39] House P.M., Holst B., Lindenau M., Voges B., Kohl B., Martens T., Lanz M., Stodieck S., Huppertz H.-J. (2015). Morphometric MRI analysis enhances visualization of cortical tubers in tuberous sclerosis. Epilepsy Res..

