# Supplementary material for: Deep Learning Approaches for Imaging-Based Automated Segmentation of Tuberous Sclerosis Complex
Source: J Clin Med. 2024 Jan 24;13(3):680. doi: 10.3390/jcm13030680 (PMC10856546; doi:10.3390/jcm13030680)
Supplement: Supplementary file 1 [file jcm-13-00680-s001.zip › jcm-2556025-supplementary.pdf]

Supplemental material

Content S1. Diagnostic criteria for tuberous sclerosis complex (TSC) 2012

Table S1. Statistic of multimodal neuroimaging characteristics of tubers across all subjects

Table S2. Statistic of multimodal neuroimaging characteristics of tubers across all subjects with several tubers

Table S3. Statistic of multimodal neuroimaging characteristics of tubers across all subjects with SEEG implantation

Table S4. Statistic summary of model performance trained by 5 different datasets

Figure S1. Visualization of intracranial tubers distribution across all 31 included patients. Blue tubers were the epileptogenic tubers, while the yellow tubers were the non-epileptogenic tubers investigated by the presurgical evaluation.

Figure S2. Multimodal quantitative neuroimaging metrics comparison of intracranial tubers across all 31 included. Detail statistical analysis was shown in **Table S3**. *: significant difference.

Reference

Content S1. Diagnostic criteria for tuberous sclerosis complex (TSC) 2012 [1]

A. Genetic diagnostic criteria<br>The identification of either a TSC1 or TSC2 pathogenic mutation in DNA from normal tissue is sufficient to make a definite diagnosis of tuberous sclerosis complex (TSC). A pathogenic mutation is defined as a mutation that clearly inactivates the function of the TSC1 or TSC2 proteins (e.g., out-of-frame indel or nonsense mutation), prevents protein synthesis (e.g., large genomic deletion), or is a missense mutation. Other TSC1 or TSC2 variants whose effect on function is less certain do not meet these criteria, and are not sufficient to make a definite diagnosis of TSC. Note that 10% to 25% of TSC patients have no mutation identified by conventional genetic testing, and a normal result does not exclude TSC, or have any effect on the use of clinical diagnostic criteria to diagnose TSC.	
B. Clinical diagnostic criteria<br>Major features<br>1. Hypomelanotic macules (≥ 3, at least 5-mm diameter)<br>2. Angiofibromas (≥ 3) or fibrous cephalic plaque<br>3. Ungual fibromas (≥ 2)<br>4. Shagreen patch<br>5. Multiple retinal hamartomas<br>6. Cortical dysplasias (* Includes tubers and cerebral white matter radial migration lines)<br>7. Subependymal nodules<br>8. Subependymal giant cell astrocytoma<br>9. Cardiac rhabdomyoma<br>10. Lymphangioleiomyomatosis (LAM)<br>11. Angiomyolipomas (≥ 2) (A combination of the two major clinical features (LAM and angiomyolipomas) without other features does not meet criteria for a definite diagnosis.)	Minor features<br>1. “Confetti” skin lesions<br>2. Dental enamel pits (> 3)<br>3. Intraoral fibromas (≥ 2)<br>4. Retinal achromic patch<br>5. Multiple renal cysts<br>6. Nonrenal hamartomas
Definite diagnosis: Two major features or one major feature with ≥ 2 minor features<br>Possible diagnosis: Either one major feature or ≥ 2 minor features	

Table S1. Statistic of multimodal neuroimaging characteristics of tubers across all subjects

Neuroimaging characteristics	Groups	Test for normal distribution		Within-group comparison	
		Lilliefors statistic	P value	Statistic	P value
Normalized tubers volume	Epileptogenic tubers	0.229	<0.001	Mann-Whitney U = 2.382	0.017
	Non-epileptogenic tubers	0.263	<0.001		
Normalized T1 signal value	Epileptogenic tubers	0.181	0.019	Mann-Whitney U = -0.264	0.792
	Non-epileptogenic tubers	0.145	0.257		
Normalized FLAIR intensity	Epileptogenic tubers	0.074	0.500	Independence Student's t = -1.082	0.285
	Non-epileptogenic tubers	0.110	0.500		
Normalized PET metabolic value	Epileptogenic tubers	0.173	0.032	Mann-Whitney U = -3.274	0.001
	Non-epileptogenic tubers	0.163	0.128		

Table S2. Statistic of multimodal neuroimaging characteristics of tubers across all subjects with several tubers

Neuroimaging characteristics	Groups	Test for normal distribution		Within-group comparison	
		Lilliefors statistic	P value	Statistic	P value
Normalized tubers volume	Epileptogenic tubers	0.251	<0.001	Paired Wilcoxon z = 1.749	0.083
	Non-epileptogenic tubers	0.263	<0.001		
Normalized T1 signal value	Epileptogenic tubers	0.168	0.104	Paired Student's t = -0.905	0.376
	Non-epileptogenic tubers	0.145	0.257		
Normalized FLAIR intensity	Epileptogenic tubers	0.106	0.500	Paired Student's t = -1.451	0.162
	Non-epileptogenic tubers	0.110	0.500		
Normalized PET metabolic value	Epileptogenic tubers	0.127	0.452	Paired Student's t = -2.502	0.021
	Non-epileptogenic tubers	0.163	0.128		

Table S3. Statistic of multimodal neuroimaging characteristics of tubers across all subjects with SEEG implantation

Neuroimaging characteristics	Groups	Test for normal distribution		Within-group comparison	
		Lilliefors statistic	P value	Statistic	P value
Normalized tubers volume	Epileptogenic tubers	0.175	0.242	Independence Student's t = 1.745	0.094
	Non-epileptogenic tubers	0.208	0.200		

Normalized T1 signal value	Epileptogenic tubers	0.130	0.500	Independence Student's *t* = -0.076	0.940
	Non-epileptogenic tubers	0.177	0.419		
Normalized FLAIR intensity	Epileptogenic tubers	0.230	0.031	Mann-Whitney *U* = -1.194	0.233
	Non-epileptogenic tubers	0.125	0.500		
Normalized PET metabolic value	Epileptogenic tubers	0.147	0.500	Independence Student's *t* = -0.988	0.333
	Non-epileptogenic tubers	0.198	0.264		

Table S4. Statistic summary of model performance trained by 5 different datasets

**Measurements**	**Test for normal distribution**										**Nonparametric Kruskal-Wallis test**	
	**Dataset 1**		**Dataset 2**		**Dataset 3**		**Dataset 4**		**Dataset 5**			
	**Lilliefors statistic**	**P value**	**Lilliefors statistic**	**P value**	**Lilliefors statistic**	**P value**	**Lilliefors statistic**	**P value**	**Lilliefors statistic**	**P value**	**Statistic**	**P value**
Accuracy	0.201	<0.001	0.199	0.001	0.201	<0.001	0.213	<0.001	0.204	<0.001	Kruskal-Wallis *H* = 0.373	0.985
Sensitivity	0.229	<0.001	0.226	<0.001	0.217	<0.001	0.230	<0.001	0.219	<0.001	Kruskal-Wallis *H* = 0.224	0.994
Precision	0.191	0.002	0.203	<0.001	0.201	0.001	0.204	<0.001	0.207	<0.001	Kruskal-Wallis *H* = 0.180	0.996
DSC	0.209	<0.001	0.207	<0.001	0.205	<0.001	0.219	<0.001	0.208	<0.001	Kruskal-Wallis *H* = 0.209	0.995

DSC: Dice similarity coefficient

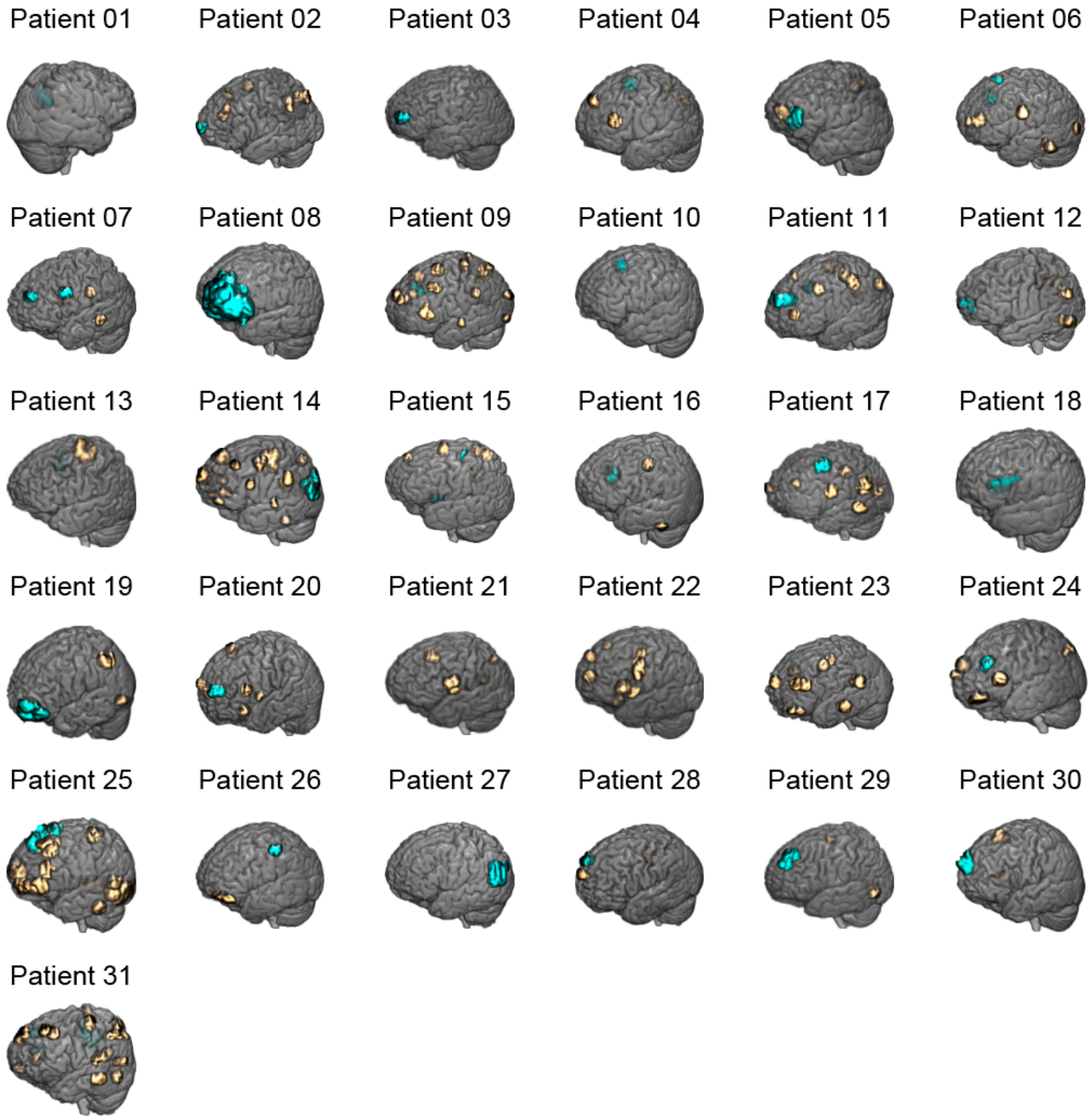

Figure S1. Visualization of intracranial tubers distribution across all 31 included patients. Blue tubers were the epileptogenic tubers, while the yellow tubers were the non-epileptogenic tubers investigated by the presurgical evaluation.

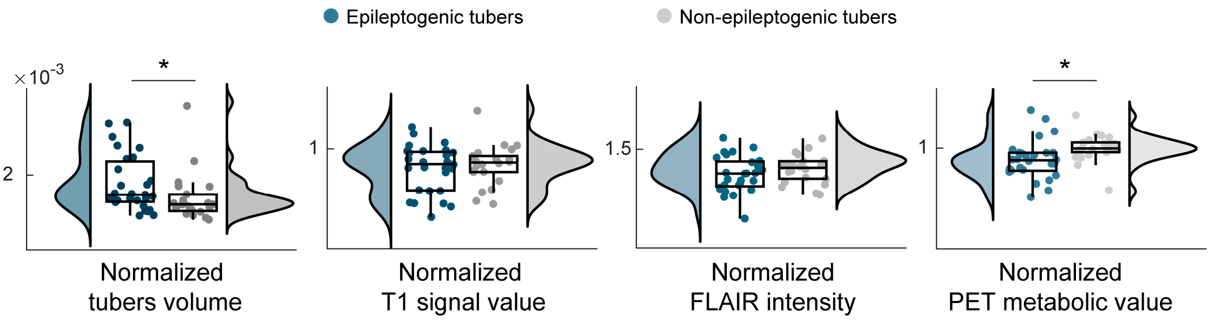

Figure S2. Multimodal quantitative neuroimaging metrics comparison of intracranial tubers across all 31 included. Detail statistical analysis was shown in **Table S1**. *: significant difference.

References

1. H. Northrup, D. A. Krueger. Tuberous sclerosis complex diagnostic criteria update: recommendations of the 2012 Iinternational Tuberous Sclerosis Complex Consensus Conference. Pediatric neurology 2013;49:243-254.
